# An integrated proteomic and glycoproteomic approach uncovers differences in glycosylation occupancy from benign and malignant epithelial ovarian tumors

**DOI:** 10.1186/s12014-017-9152-2

**Published:** 2017-05-10

**Authors:** Qing Kay Li, Punit Shah, Yuan Tian, Yingwei Hu, Richard B. S. Roden, Hui Zhang, Daniel W. Chan

**Affiliations:** 1 Department of Pathology, The Johns Hopkins University School of Medicine, Baltimore, MD 21287 USA; 2 Department of Pathology, The Johns Hopkins Bayview Medical Center, 4940 Eastern Ave., Building AA, Room 154B, Baltimore, MD 21224 USA

**Keywords:** Ovarian high-grade serous carcinoma, Proteomics, Glycoproteomics

## Abstract

**Background:**

Epithelial ovarian carcinomas encompass a heterogeneous group of diseases with a poor 5-year survival rate. Serous carcinoma is the most common type. Most FDA-approved serum tumor markers are glycoproteins. These glycoproteins on cell surface or shed into the bloodstream could serve as therapeutic targets as well as surrogates of tumor. In addition to glycoprotein expressions, the analysis of protein glycosylation occupancy could be important for the understanding of cancer biology as well as the identification of potential glycoprotein changes in cancer. In this study, we used an integrated proteomics and glycoproteomics approach to analyze global glycoprotein abundance and glycosylation occupancy for proteins from high-grade ovarian serous carcinoma (HGSC) and serous cystadenoma, a benign epithelial ovarian tumor, by using LC–MS/MS-based technique.

**Methods:**

Fresh-frozen ovarian HGSC tissues and benign serous cystadenoma cases were quantitatively analyzed using isobaric tags for relative and absolute quantitation for both global and glycoproteomic analyses by two dimensional fractionation followed by LC–MS/MS analysis using a Orbitrap Velos mass spectrometer.

**Results:**

Proteins and *N*-linked glycosite-containing peptides were identified and quantified using the integrated global proteomic and glycoproteomic approach. Among the identified *N*-linked glycosite-containing peptides, the relative abundances of glycosite-containing peptide and the glycoprotein levels were compared using glycoproteomic and proteomic data. The glycosite-containing peptides with unique changes in glycosylation occupancies rather than the protein expression levels were identified.

**Conclusion:**

In this study, we presented an integrated proteomics and glycoproteomics approach to identify changes of glycoproteins in protein expression and glycosylation occupancy in HGSC and serous cystadenoma and determined the changes of glycosylation occupancy that are associated with malignant and benign tumor tissues. Specific changes in glycoprotein expression or glycosylation occupancy have the potential to be used in the discrimination between benign and malignant epithelial ovarian tumors and to improve our understanding of ovarian cancer biology.

**Electronic supplementary material:**

The online version of this article (doi:10.1186/s12014-017-9152-2) contains supplementary material, which is available to authorized users.

## Background

Ovarian cancer is the fifth most common cancer in women in the US, with 22,440 new cases diagnosed and more than 14,080 cancer-related deaths in 2017 [1]. Approximately 80% of patients presented with locally advanced and/or metastatic disease at the time of diagnosis and only ~40% survive five years, although early stage disease can be treated successfully [1, 2]. The poor outcome of patients has not changed much in the past three decades, despite advances in surgery and chemotherapies and major efforts to develop a screening test with greater predictive value than a standalone serum test for the tumor-shed glycoprotein CA-125, which is FDA approved for monitoring therapy. The current largest and most promising clinical screening trial, the UK Collaborative Trial of Ovarian Cancer Screening (UKCTOCS), which includes 202,638 women over 14-year period, has suggested for the first time that a multimodal screening approach with repeat serum CA-125 testing and transvaginal ultrasonography could potentially reduce the mortality; however, it is still not yet conclusive [3–5]. There remains a need to find better methods that can be use in screening for ovarian cancer while it is still curable with conventional therapy.

Ovarian carcinoma is a heterogeneous disease with various histological subtypes, making the development of a single biomarker challenging and suggesting focus on the most impactful subtype is warranted [6–12]. Most ovarian cancers are epithelial in origin, with high grade serous carcinomas accounting for 70–80% of cases and the rarer subtypes, including clear cell (3%), endometrioid (<5%), mucinous (<3%) carcinoma and others [2, 8–12]. Each histological subtype is now being considered separate disease with characteristic cytogenetic features, molecular signatures, oncogenic signaling pathways and clinical/biological behaviors [8–12]. However, based on clinical characteristics, histological patterns and molecular signatures, ovarian cancer can be divided into two types—type I, which comprises endometrioid, clear cell, mucinous, and low-grade serous cancer, and type II, which comprises high-grade serous carcinoma (HGSC), undifferentiated carcinomas and carcinosarcoma [2, 8–12].

Type I ovarian carcinomas are considered to arise via a well-defined adenoma-carcinoma sequence from a benign precursor lesion, to evolve into a malignant lesion with a stepwise fashion [2, 8–12]. Type I carcinomas are usually slow growing neoplasms with indolent clinical behavior. They frequently exhibit molecular abnormalities in several genes, such as somatic mutations of *CTNNB1* (Catenin (Cadherin-Associated Protein), Beta 1), *PTEN* (phosphatase and tensin homolog) and *PIK3CA* (phosphatidylinositol-4,5-bisphosphate 3-kinase, catalytic subunit α) in endometrioid carcinomas; *KRAS* (Kirsten rat sarcoma viral oncogene homolog) mutations in mucinous carcinomas; *PIK3CA* activating and *ARID1A* inactivating mutations in clear cell carcinomas, and others. By contrast, type II carcinomas originate from the fimbriated end of the fallopian tube and possibly ovarian surface epithelia, develop rapidly and behave aggressively [8–12]. Mutations in TP53 (>90%) are common in type II carcinomas [8–12]. Although it is well-known that different histomorphological ovarian carcinomas correlate with characteristic molecular features and clinical behavior, protein and glycosylation signatures of ovarian carcinoma are still not well-studied.

At cellular level, the majority of proteins either secreted from cells or located on extracellular surface are glycoproteins. Glycoproteins play important roles in the regulation of cellular functions, including cell differentiation, proliferation, cellular interactions with their surrounding environment, and invasion or metastasis of tumor cells [13]. In addition, most FDA approved serum tumor markers are glycoproteins, such as carcinoembryonic antigen (CEA), prostate specific antigen (PSA), CA125, CA19-9 [14]. Therefore, it is important to analyze glycoprotein signatures of ovarian cancer tissue, which may provide critical information for the discovery of tumor-associated proteins. Furthermore, our previous studies have shown that the glycosylation pattern of glycoproteins is also associated with biological characteristics of the cancer [15–17]. Similarly, in ovarian cancer changes of glycoproteins may be a result of changes in protein concentration and/or protein glycosylation occupancy [18–21]. Therefore, it is necessary not only to study the global protein profile, but also the glycoprotein profile in ovarian cancers in the search of novel glycoprotein changes.

Quantitative proteomic analysis of several types of ovarian tumors have demonstrated different protein profiles in ovarian cancers [22–28]. Our recent study of 177 HGSCs has demonstrated characteristic protein profiles in ovarian carcinoma tissues and provided an updated knowledge of the proteomics of ovarian cancer [7]. This large scale of study also uncovers the characteristic phosphoproteins of the HGSC. However, the understanding of protein signature, particularly the profile of glycoprotein in HGSC, is still suboptimal.

In this study, we investigated the global proteome and glycoproteome profiles associated with HGSC and benign serous cystadenoma using quantitative LC–MS/MS-based global proteomics and glycoproteomics approach, to simultaneously identify and quantify global proteome and glycoproteome by iTRAQ labeling and LC–MS/MS; and compared the relative abundance of glycoproteins as well as the change in relative glycosylation occupancy of that protein in malignant versus benign ovarian tumor tissues.

## Methods

### Collection of ovarian tumor and benign tissues

Fresh-frozen tissues of three HGSC (stage IIIc from white females) and three benign serous cystadenoma (from white females) were included. All tissues were stored at −80 °C until use. All diagnoses were rendered by the American Pathology Medical Board certified surgical pathologists based on the H&E (hematoxylin and eosin) stained sections. This study was approved by the Johns Hopkins Medical Institution Review Board (IRB).

### Reagents

Hydrazide resin (Bio-Rad, Hercules, CA), sodium periodate (Bio-Rad, Hercules, CA), tris (2-carboxyethyl) phosphine (TCEP) (Pierce, Rockford, IL), PNGase F (New England Biolabs, Ipswich, MA), sequencing grade trypsin (Promega, Madison, WI), C18 columns (Waters, Sep-Pak Vac) were used in our experiment. Additional lab supply and chemicals were purchased from Sigma-Aldrich. iTRAQ reagent and mass calibration standards were from AB SCIEX (Foster City, CA); BCA assay kit was from Pierce (Rockford, IL).

### Peptide extraction and glycopeptide enrichment

**Fig. 1:**
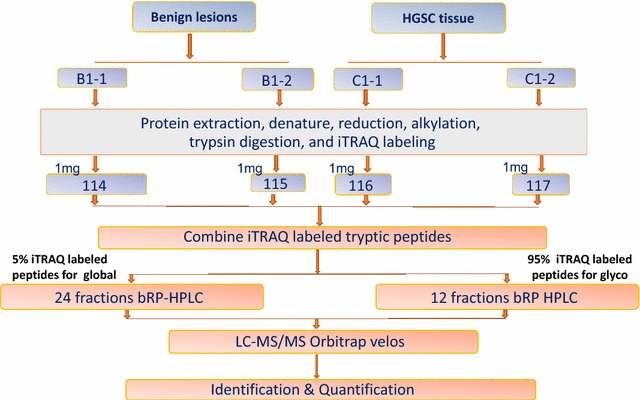
The strategy of multiplex identification of global proteins and *N*-glycoproteins in HGSC

The tissues were homogenized on ice to extract proteins. The protein concentration was measured by BCA assay. From each tissue, at least 2 mg proteins were digested with trypsin at a ratio of 1:50 (w/w, enzyme:protein) in trypsin digestion buffer (100 mM Tris–HCl, pH 7.5) at 37 °C overnight with gentle shaking. Peptides were cleaned using a C18 column and the concentration was determined by BCA assay. Same amount of the peptide from each sample were labeled by iTRAQ-4plex according to manufacturer’s instruction. For the first iTRAQ experiment, 1 mg of proteins of a benign tissue was labeled with report ion 114 and 115, whereas, the same amount of protein from a malignant sample was labeled with report ions 116 and 117 for technical replicate (Fig. 1). For the second iTRAQ experiment, 1 mg of proteins from the second benign sample was labeled with report ion 114, and the third benign sample was labeled with report ion 115, whereas the second tumor tissue was labeled with repot ion 116 and the third tumor sample was labeled with 117. After iTRAQ labeling, 4 labeled samples from each iTRAQ set was combined, and 200 µg of labeled peptides were used for global proteomics analysis. The rest of 3.8 mg of iTRAQ labeled peptides were enriched for *N*-linked glycopeptides using the solid phase extraction of glycopeptides (SPEG) method previously descripted [29, 30]. Briefly, peptides were oxidized by 10 mM sodium periodate (Bio-Rad, Hercules, CA) at room temperature for 1 h. Glycopeptides were covalently conjugated to a solid support via hydrazide chemistry (Bio-Rad, Hercules, CA), with incubation at room temperature overnight. The hydrazide beads were washed with 1.5 M NaCl, and water prior to release of formerly *N*-linked glycopeptides from solid support by PNGase F at 37 °C overnight. The eluent was purified by Sep-Pak Vac C18 cartridge and resuspended in 40 µL of 0.4% acetic acid.

### Fractionation by basic reverse liquid chromatography (*bRPLC*)

Labeled peptides were fractionated offline using bRPLC as previously reported [31, 32]. Briefly, approximately 100 µg of 4-plex iTRAQ labeled tryptic peptides or 10 µg of labeled glycopeptides were separated on a reversed phase Zorbax extend-C-18 column (4.6 × 100 mm column containing 1.8-µm particles) from Agilent Technology (Santa Clara, CA) using an Agilent 1220 Infinity HPLC System. The solvent consisted of 10 mM ammonium formate (pH 10) as mobile phase A and 10 mM ammonium formate and 90% ACN (pH 10) as mobile phase B. The separation gradient was set as follows: 2% B for 10 min, from 2 to 8% B for 5 min, from 8 to 35% B for 85 min, from 35 to 95% B for 5 min, and 95% B for 25 min. Ninety-six fractions were collected across the LC separation and were concatenated into 24 fractions for tryptic peptides and 12 fractions for glycopeptides (Fig. 1). The samples were dried in a Speed-Vac and stored at −80 °C until LC–MS/MS analysis.

### LC–MS/MS analysis

Fractionated iTRAQ labeled peptides were analyzed on a LTQ Orbitrap Velos mass spectrometer (Thermo Fisher Scientific Inc., Rockford, IL). 1 µg of peptides per injection was loaded on a C18 column (75 µm ID × 15 cm, C18 5 µm, 100 A), and gradient eluted over 100 min at 300 nL/min into the mass spectrometer. The HPLC mobile phase A and B were 0.2% formic acid in HPLC grade water and 0.2% formic acid in HPLC grade acetonitrile, respectively. The mobile phase B was increased from 5 to 40% in 90 min. LC–MS/MS data was obtained using a data dependent analysis of the top ten precursors and a dynamic exclusion of 30 s.

### Data analysis

The acquired LC–MS/MS data was searched against the Homo sapiens taxonomy of the IPI Human database V3.87 using Sequest (search algorithm within Proteome Discoverer, Thermo Scientific version 1.3) with following parameters: two missed cleavages allowed, trypsin as cleavage enzyme, a tolerance of 15 ppm on precursors and 0.06 Daltons on the fragment ions. Modifications allowed include: carbamidomethylation of cysteines sets to static, iTRAQ4plex on N-Terminus sets to static, iTRAQ4plex on lysine sets to variable, oxidation of methionine and deamination of asparagine and glutamine set to variable. Data was also searched against a decoy database and filtered with a 1% false discovery rate (FDR).

Glycopeptides were further filtered for consensus sequence of *N*-linked glycosylation motif NXS/T. The Relative ratios of abundance of *N*-glycoprotein between cancer and benign samples were calculated.

## Results and discussion

### Detection of glycoprotein and glycosylation occupancy changes in the HGSC tissue

We first analyzed the first iTRAQ set to identify the proteins and glycoproteins from malignant and benign ovarian tumor tissues in replicate analyses. The overall analytic strategy is schematically illustrated in Fig. 1. It consists of following steps: (1) recover and iTRAQ labeling of peptides from tumor tissue and benign ovarian lesions, (2) combine iTRAQ labeled peptides, (3) simultaneously analyze global and *N*-glycopeptides in conjunction with extensive fractionation and high-resolution tandem mass spectrometry, (4) analyze and compare profiles of global protein and glycoproteins between HG serous carcinoma and benign lesions. By using this approach, we were able to identify the changes in global proteins and glycoproteins simultaneously.

The iTRAQ technique was used to allow for quantitation in conjunction with extensive fractionation using bRPLC and high-resolution tandem mass spectrometry to provide in-depth of coverage for peptide and protein identification and quantification. A total of 4817 proteins were identified and quantified (Additional file 1: Table S1). Using glycoproteomic analysis, we identified and quantified 1629 unique *N*-linked glycosite-containing peptides (Additional file 2: Table S2).

The relative abundances of global proteins and glycosite-containing peptides in tumor tissues were summarized in Additional file 1: Table S1 and Additional file 2: Table S2. Among them, 36 *N*-linked glycosite-containing peptides showed more than threefold increased levels in the HGSC tissue cancerous tissue comparing to benign tissue (Table 1). In the experiment, isobaric labeling was performed prior to split a sample for both proteomic and glycoproteomic analysis. This technique allowed us to determine the level of proteomic change as well as the level of glycoprotein change, simultaneously. Based upon the technique, we first determined changes of in glycosite-containing peptides and protein abundances, and then, we correlated them with changes of glycosite-containing peptides to global protein levels to provide the relative differences in glycosylation occupancy. We found that 10 glycosite-containing peptides were increased in glycosylation without significant changes in global protein levels (Table 2). These glycoproteins are folate receptor alpha, Golgi apparatus protein 1, CD 180 antigen, leucine-rich repeats and immunoglobulin-like protein 1, Cell adhesion molecule 1, 4F2 cell-surface antigen heavy chain, integrin alpha-X, ADP-ribosyl cysclase/cyclic ADP-ribose hydrolase 1, and semaphoring-4B.

**Table 1:** Thirty-six glycosite-containing peptides with threefold increased levels in ovarian tumor comparing to benign tumors using glycoproteomic analysis

Description	Sequence	Glycosite average
Folate receptor alpha	gWnWTSGFNk	12.04
Sialate *O*-acetylesterase	aLAYGEknLTFEGPLPEkIELLAHk	9.26
CD180 antigen	lmnLTFLDLTR	7.70
Toll-like receptor 7	dAFLnLTk	7.69
Prolyl endopeptidase FAP	fmGLPTkDDNLEHYknSTVmAR	5.62
CD48 antigen	vQkEDnSTYImR	5.52
Prolyl endopeptidase FAP	vQnVSVLSIcDFR	5.44
Tetraspanin-15	nTTEVVNTmcGYk	5.31
Lysosome-associated membrane glycoprotein 3	yFNIDPnATQASGNcGTR	5.07
Prolyl endopeptidase FAP	dILnGTFSYk	4.88
Folate receptor α	kNAccSTnTSQEAHk	4.35
Prostatic acid phosphatase	vYDPLYcESVHnFTLPSWATEDTmTk	4.28
Integrin beta-6	eVEVnSSk	4.28
Alpha-galactosidase A	lGIYADVGnk	4.02
Nuclear pore membrane glycoprotein 210	eGSGYFFLnTSTADVVk	3.99
Semaphorin-4B	fEAEHISnYTALLLSR	3.92
CUB domain-containing protein 1	eSnITVLIk	3.83
Endoplasmin (94 kDa glucose-regulated protein)	eLISnASDALDk	3.83
ADP-ribosyl cyclase/cyclic ADP-ribose hydrolase 1	fAEAAcDVVHVmLnGSR	3.80
Sialate *O*-acetylesterase	nLTFEGPLPEkIELLAHk	3.77
CUB domain-containing protein 1	tcSSnLTLTSGSk	3.68
Extracellular sulfatase Sulf-1	dYFTDLITnESINYFk	3.66
Procollagen-lysine, 2-oxoglutarate 5-dioxygenase 2	eAInITLDHk	3.63
Golgi apparatus protein 1	nDTLQEAk	3.60
Glutamate carboxypeptidase 2	nFTEIASk	3.51
Leucine-rich repeats and immunoglobulin-like domains protein 1	sLnLSYNk	3.49
4F2 cell-surface antigen heavy chain	dASSFLAEWQnITk	3.49
Sortilin isoform X1	nFkDITDLInNTFIR	3.47
Integrin alpha-X	yLnFSESEEk	3.47
Amiloride-sensitive amine oxidase	dcSmPPPFSYnGTYRPV	3.38
Tetraspanin-13	sVNPnDTcLAScVk	3.31
Desmocollin-2	anYTILk	3.30
Cell adhesion molecule 1	fQLLnFSSSELk	3.24
NHL repeat-containing protein 3	lSQDFmILWLHGEnGTGPAk	3.12
Apolipoprotein(a)	wEYcnLTR	3.07
Prosaposin	nSTkQEILAALEk	3.01

**Table 2:** Unique glycosylation occupancy changes in 10 glycosite-containing peptides

Description	Sequence	Glycosite average	Global average	Glycosite/global
Folate receptor alpha	gWnWTSGFNk	12.04	1.97	6.10
Golgi apparatus protein 1	nDTLQEAk	3.60	0.63	5.69
CD180 antigen	lmnLTFLDLTR	7.70	1.46	5.27
Leucine-rich repeats and immunoglobulin-like domains protein 1	sLnLSYNk	3.49	0.93	3.77
Cell adhesion molecule 1	fQLLnFSSSELk	3.24	1.19	2.73
4F2 cell-surface antigen heavy chain	dASSFLAEWQnITk	3.49	1.38	2.53
Integrin alpha-X	yLnFSESEEk	3.47	1.37	2.53
Folate receptor α	kNAccSTnTSQEAHk	4.35	1.97	2.20
ADP-ribosyl cyclase/cyclic ADP-ribose hydrolase 1	fAEAAcDVVHVmLnGSR	3.80	1.81	2.10
Semaphorin-4B isoform 1	fEAEHISnYTALLLSR	3.92	1.88	2.09

Proteins and glycosite-containing peptides identified from the second iTRAQ experiment with 2 additional benign and 2 additional HGSC tumors were further analyzed to obtain quantitative proteomic and glycoproteomic data to determine the change in each *N*-linked glycosite and corresponding changes in glycoprotein level. A total of 5183 proteins and 1853 *N*-linked glycosite-containing peptides were identified and quantified (Additional file 3: Table S3; Additional file 4: Table S4). From these data, the changes in glycosite-containing peptides and global proteins in additional 2 cases of HGSC and 2 benign tumors were quantified. This allowed the determination of the changes of the glycosylation occupancies at each glycosite from glycoproteins in HGSC and benign cases. Besides the well-known issue of missing data in the two different iTRAQ experiments by tandem mass spectrometry, which resulted in different glycosite and protein identifications, we also observed individual variation in glycosylation occupancies in HGSC and benign cases. A large number of sample size is needed to determine the disease-associated changes in glycosylation occupancies versus individual variation.

### Merit of current techniques in the detection of glycoproteins

In the past decade, several highly sensitive proteomic techniques, such as high-content quantitative proteomic using LC–MS/MS, SPEG, and iTRAQ labeling have been developed [29–32]. These techniques have an increased sensitivity and throughput capability of accurate analysis of biological samples. The combination of these techniques has tremendously improved our ability for the detection of low abundance proteins in biological samples.

By using these advanced techniques, several recent publications have shown that different histological type of ovarian cancer are associated with distinct type of protein profiles in both tumor tissue and serum samples, including changes of glycoproteins. Abbott et al. [18] studied tumor-specific glycan changes between tumor and normal ovarian tissue and identified glycoproteins markers that show tumor-specific glycosylation changes. Shetty et al. [19] identified 10 *N*-linked sialylated glycopeptides significantly upregulated in ovarian cancer patients’ serum samples. Kuzmanov et al. [20] discovered 13 sialoglycopeptides in ovarian cyst fluid and ascites fluid of ovarian cancer patients. Using SELDI-TOF–MS platform, Zhang et al. [33, 34] identified differential expressions of several serum proteins in ovarian cancers; and later they developed a FDA-approved multivariate index assay, OVA1 test, including five biomarkers: apolipoprotein A1, transthyretin, transferrin, β-2 microglobulin, and CA125 [35]. The OVA1 and second generation OVA2 tests are currently used to assist in physicians to triage women with suspected pelvic masses. This is important because surgery on ovarian cancer is associated with improved outcomes if performed by a specialist gynecologic oncologist and this test can be used to help triage patients. However, these tests lack the predictive value for routine screening and the knowledge of molecular protein signature of ovarian cancers, particularly HGSC, is still suboptimal.

The glycoproteome of tumor tissue is complex and consists of both high and low abundance glycoproteins. The detection of low abundance glycoproteins is notoriously difficult, due to the obscuring artifact of high abundance glycoproteins. In this study, we performed the isobaric labeling prior to split the samples for proteome and glycoproteome analyses, therefore, we were able to acquire glycopeptide abundance and correlate them with changes of the parent protein abundance to provide the net differences in glycosylation. There are several advantages of our strategy of focusing on the identification of *N*-glycoprotein changes in protein expression and glycosylation occupancy using the integrated global proteomics and glycoproteomics approach. First, the combination of iTRAQ labeling with extensive fractionation and high performance reversed phase liquid chromatography and high-resolution tandem mass spectrometry can provide high depth of coverage for peptide and protein identification for global proteomics. Second, the SPEG glycopeptides-capturing technique allows the identification and quantification of glycosite-containing peptides from extracellular tumor-derived or secreted proteins in tumor tissues. Finally, the integrated proteomic and glycoproteomic analyses of iTRAQ labeled prior to the capture of glycosite-containing peptides can relate glycopeptide abundance changes in parent protein abundance to provide the net differences in glycosylation. Our approach allows us to identify unique *N*-glycoproteins whose changes may not be found at global protein levels but can only be identified by glycan levels. Thus, we not only identified *N*-glycoprotein profile, but also compared *N*-glycoprotein profile with global protein profile in tumor tissues. However, the small sample size in our study is a limitation. A larger scale of study is necessary to further validate our findings.

### Significance of our findings and further direction

HGSC is characterized by mutations of *TP53*, extensive gene copy number alterations (CNAs), alterations in homologous recombination (HR) and aberrations in certain pathways such as PI3 Kinase, RAS, Notch, FOXM1, and RB1 signaling/cell cycle control [2, 8–11]. In our first iTRAQ experiment, among 1629 glycosite-containing peptides, 36 glycosite-containing peptides with more than threefold increased were identified, respectively. In addition, 10 glycosite-containing peptides revealed increase in glycosylation levels, but without a corresponding change at protein levels. We found unique changes of glycosylation occupancy in these glycoproteins, in comparison to changes of their protein levels. Our findings indicated that the unique glycoprotein occupancy might be specific to each glycoprotein. Our findings need to be further verified in a larger scale of study.

These glycoproteins have been demonstrated to play different biological role in ovarian cancers. For example, cell adhesion molecule I plays critical roles in tumor progression [36]. Folate receptor (FOLR) has also been detected in 78% of HGSC by others, and it has been related by an increased overall survival in ovarian cancers [37]. Interestingly, we also detected the expression of CD180. CD180 is a related member of the Toll-like receptor family, and its expression has been related to tumor stage and progression [38]. Taken together, our study provides evidence that multiplex analysis of tumor tissues could provide important insight for understanding the molecular protiome signature. Our quantitative proteomic analysis sheds a light on the characteristics of the complex (glycol) proteome of ovarian tumors and provides an evidence of tumor-associated protein in HGSC. Further study is necessary to improve our current knowledge in the fields of ovarian cancer.

Finally, an elevated level of ADP-ribosyl cyclase/cyclic ADP-ribose hydrolase 1 (member of CD157 pathway), was identified in our study. Both ADP-ribosyl cyclase and CD157 belongs the ADP-ribosyl cyclase gene family. They play important roles in the regulation of cancer cell invasion and progression [39–43]. Several previous publications indicate that CD157 is overexpressed and functions as an ectoenzyme and receptor in ovarian cancer cells [39–43]. Furthermore, CD157 regulates the interaction among tumor cells and the expression of extracellular matrix proteins. Taken together, our finding is consistent with prior reports that the ADP-ribosyl cyclase/CD157 pathway plays a critical role in the regulation of ovarian cancer cell invasion and progression.

## Conclusion

In summary, with this comprehensive study of proteome and glycoproteome of ovarian HGSC and benign tissues, we were able to determine not only changes of protein concentrations in global proteome and glycoproteome, but also changes in relative glycosylation occupancy at specific glycosylation sites of the protein. It is important to recognize that certain proteomic changes can only be demonstrated at glycosylation occupancy levels. These types of changes could be easily missed by studies which might only focus on the quantification determination of total glycoprotein or at glycosite levels. The glycosylation occupancy of cancer cell may play an important role in understanding of the biology of ovarian cancers as well as in serving as potential biomarkers for ovarian cancer patients.

## Additional files

**Additional file 1: Table S1.** Global proteomic analysis of ovarian tissue proteins from the first iTRAQ experiment.

**Additional file 2: Table S2.** Glycosite-containing peptides identified from glycoproteomic analysis from benign and HGSC tissues in iTRAQ experiment 1.

**Additional file 3: Table S3.** Proteomic analysis from additional 2 benign and 2 HGSC tissues in iTRAQ experiment 2.

**Additional file 4: Table S4.** Glycosite-containing peptides identified from glycoproteomic analysis from additional 2 benign and 2 HGSC tissues in iTRAQ .
